# Sustained Isoprostane E2 Elevation, Inflammation and Fibrosis after Acute Ischaemia-Reperfusion Injury Are Reduced by Pregnane X Receptor Activation

**DOI:** 10.1371/journal.pone.0136173

**Published:** 2015-08-24

**Authors:** Aimen O. Amer, Philip M. Probert, Michael Dunn, Margaret Knight, Abigail E. Vallance, Paul A. Flecknell, Fiona Oakley, Iain Cameron, Steven A. White, Peter G. Blain, Matthew C. Wright

**Affiliations:** 1 Institute of Cellular Medicine, Newcastle University, Newcastle, United Kingdom; 2 Medical Toxicology Centre, Newcastle University, Newcastle, United Kingdom; 3 Comparative Biology Centre, Newcastle University, Newcastle, United Kingdom; 4 Department of Pathology, Altnagelvin Hospital, Londonderry, United Kingdom; University of Navarra School of Medicine and Center for Applied Medical Research (CIMA), SPAIN

## Abstract

Liver grafts donated after cardiac death are increasingly used to expand the donor pool but are prone to ischaemic-type biliary lesions. The anti-inflammatory effects of the activated pregnane X receptor have previously been shown to be beneficial in a number of inflammatory liver conditions. However, its role in reducing peri-portal inflammation and fibrosis following ischaemia-reperfusion injury has not been investigated. Hepatic injury and its response to pregnane X receptor activation was examined after partial hepatic ischaemia-reperfusion injury induced by surgically clamping the left and middle lobar blood vessels in rats. Molecular and pathological changes in the liver were examined over the following 28 days. Ischaemia-reperfusion injury resulted in transient cholestasis associated with microvillar changes in biliary epithelial cell membranes and hepatocellular injury which resolved within days after reperfusion. However, in contrast to chemically-induced acute liver injuries, this was followed by sustained elevation in isoprostane E2, peri-portal inflammation and fibrosis that remained unresolved in the ischaemic reperfused lobe for at least 28 days after clamping. Administration of pregnenolone-16α-carbonitrile—a rodent-specific pregnane X receptor activator—resulted in significant reductions in cholestasis, hepatic injury, ischaemic lobe isoprostane E2 levels, peri-portal inflammation and fibrosis. Hepatic ischaemia-reperfusion injury therefore results in inflammatory and fibrotic changes that persist well beyond the initial ischaemic insult. Drug-mediated activation of the pregnane X receptor reduced these adverse changes in rats, suggesting that the pregnane X receptor is a viable drug target to reduce ischaemic-type biliary lesions in recipients of liver transplants donated after cardiac death.

## Introduction

The growth in liver transplant waiting lists is currently being met with only a modest increase in the number of organ donors and the gap between organ demand and supply continues to widen [1,2]. Organs donated after cardiac death (DCD) are increasingly being utilised in an attempt to bridge this gap [3] despite a significantly higher risk of biliary complications and graft failure among recipients of these organs compared to those donated after brain death (DBD) [4,5]. Ischaemic-type biliary lesions (ITBL) represent a substantial proportion of complications and are a significant cause of graft failure and death following deceased donor liver transplantation [6–8]. Ischaemia and the inflammatory cascade subsequent to reperfusion are thought to play a major role in the pathogenesis of these lesions, which usually present as biliary strictures several months after transplantation [7]. Given the inherently long exposure of DCD organs to warm ischaemia, these organs are particularly prone to ITBL [5,8]. Nevertheless, while the rate of DBD organs has plateaued over the past decade, the contribution of DCD organs to the deceased donor pool has risen annually [2]. In view of the existing organ shortage and the significant risk associated with DCD liver transplantation, it is imperative that all potential strategies for optimising DCD grafts are examined in order to minimise graft loss and insure the best possible outcome for liver transplant recipients.

The pregnane X receptor (PXR) is a promising drug target for the treatment of inflammatory liver disease [9–13]. The PXR is a member of the nuclear receptor gene superfamily of ligand-activated transcription factors [14]. It is expressed at relatively high levels in hepatocytes and is activated by several drugs (e.g. cyclosporin and rifampicin) and endobiotics (e.g. bile acids) [11,15]. In its activated form, the PXR promotes the metabolism, transport and excretion of toxic compounds through induction of the Cyp3a subfamily of cytochromes P450 and modulation in the expression of transporters (Na+-taurocholate cotransporting polypeptide and solute carrier organic anion transporter family, member 1B1) [15,16]. PXR activation has also been shown to have an anti-inflammatory effect and has been explored as a promising therapeutic target in inflammatory bowel disease [17–19]. Several groups have demonstrated that PXR activation promotes rodent hepatocyte growth in-vivo [20,21], although recent work has suggested that PXR activation alone has an hypertrophic effect with hyperplasia dependent on CAR activation or PPARa activation in rodent liver [22] Our group has shown that PXR agonists significantly reduce NF-κB-induced peri-portal inflammation and fibrosis [9–11] and recently, this has been extended to show that PXR activation directly inhibits inflammatory responses in hepatocytes [23].

We hypothesised that drug-mediated PXR activation will have a significant beneficial impact on liver function after IRI through its growth-promoting, anti-inflammatory and anti-fibrotic effects and its promotion of endobiotic excretion. To test this hypothesis, the effect of PXR activation in a rat model of hepatic ischaemia-reperfusion injury was examined. The data in this paper show for the first time that a single acute ischaemia results in progressive elevation of a specific isoprostane, peri-portal inflammation and fibrosis that persists for at least 28 days after injury. Furthermore we demonstrate that activation of the PXR reduces isoprostane elevation, injury, inflammation and fibrosis, therefore identifying a realistic clinical drug target to reduce ITBL in transplanted livers.

## Materials and Methods

### Materials

Pregnenolone-16α-carbonitrile (PCN) is a rodent-specific PXR activator [9] and was purchased from Sigma Aldrich (St Louis, MI, USA) along with dimethyl sulfoxide (DMSO) used as solvent vehicle. Cell lysis buffer was obtained from New England Biolabs (Hitchin, UK). DAB (3,3-diaminobenzidine) substrate-chromogen kit was purchased from Dako (Carpinteria, CA, USA) in addition to polyclonal goat anti-mouse and anti-rabbit HRP antibodies. Rabbit monoclonal antibody for vimentin and mouse monoclonal antibody for Cyp3a1 were obtained from Abcam (Cambridge, UK) whereas mouse monoclonal antibody for β-actin was purchased from Sigma Aldrich. Pierce enhanced chemi-luminescence (ECL) substrate kit was purchased from Thermo Scientific (Rockford, IL, USA). Authentic isoprostanes (F2, E2/D2 and A2/J2) were purchased from Cambridge Biosciences. Gel electrophoresis was performed using NuPAGE reagents and 4–12% bis-tris precast gels, and the iBlot dry blotting system was used for Western blotting (Life Technologies, Paisley, UK). Malondialdehyde bis(dimethyl acetal) (MDA), thiobarbituric Acid (TBA) and butylated hydroxytoluene (BHT) were all purchased from Sigma Aldrich. Hyaluronan Quantikine ELISA kit was purchased from R&D Systems (Minneapolis, MN, USA). TRIzol reagent was purchased from Life Technologies. RQ1 RNase-free DNase I, M-MLV Reverse transcriptase, Random Primers, dNTP Mix and Pfu DNA Polymerase were all purchased from Promega (Southampton, UK). SYBR Green Jumpstart TaqReadyMix was purchased from Sigma Aldrich as were all gene-specific primers. MicroAmp Fast Optical 96-Well PCR Reaction plates were purchased from Applied Biosystems (Foster City, CA, USA). Primers were designed using NCBI Primer-Blast (Bethesda, MD, USA). Unless otherwise specified, all other products were purchased from Sigma Aldrich and all purchased reagents were of the highest commercially available purity.

### Animals

Male Sprague Dawley rats (Charles River, Margate, UK), weighing 350-500g were used in all studies. Animals were housed in light- and temperature-controlled conditions and allowed water and standard pelleted chow ad libitum. Animal Welfare and Ethical Review Body (Newcastle University) and UK Home Office approval were obtained for animal procedures. All experiments were carried out in accordance with the Animals (Scientific Procedures) Act 1986 and in strict compliance with other local and national guidelines and policies.

### In-vivo ischaemia reperfusion injury model

**Fig 1:**
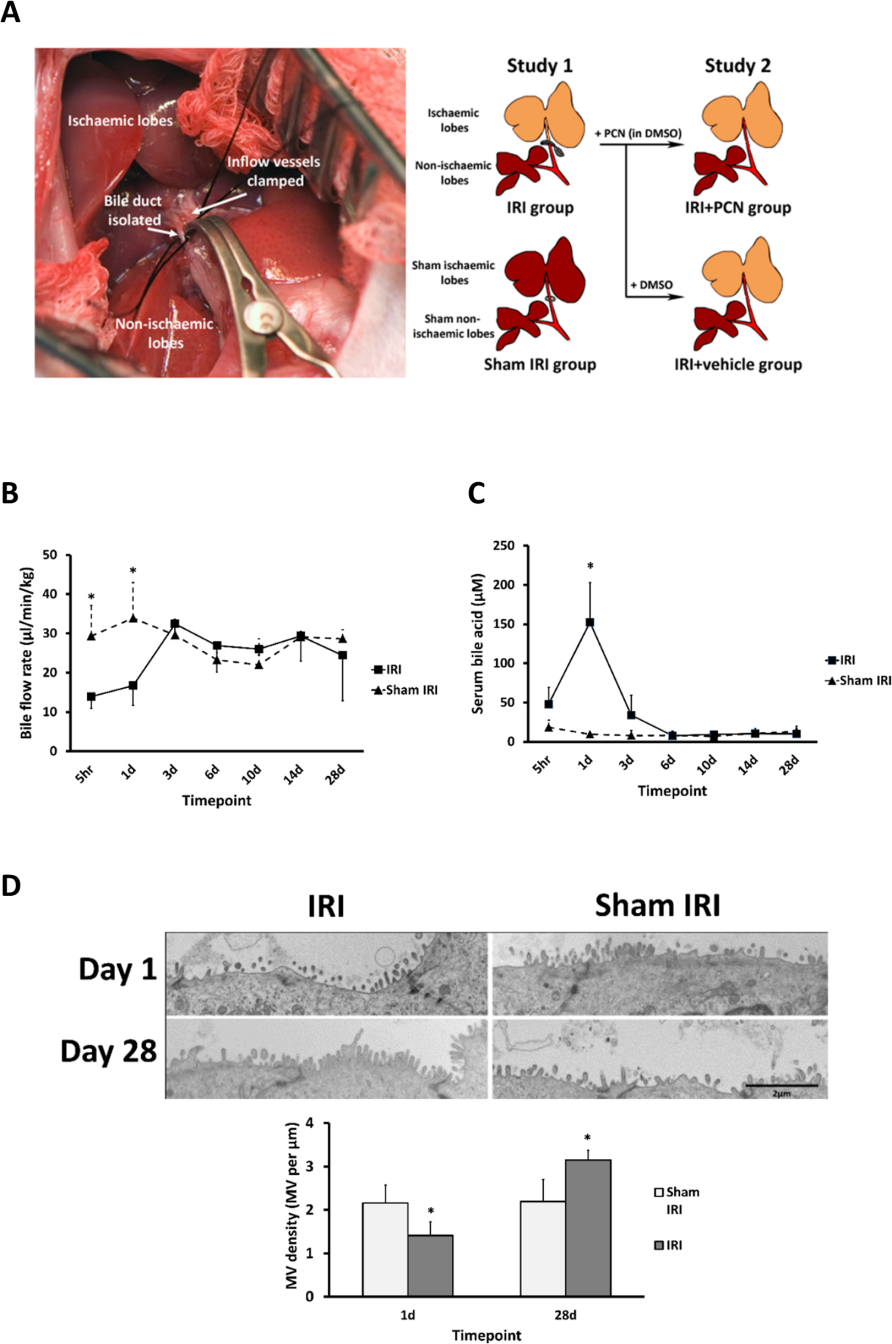
IRI results in a transient cholestasis and altered biliary physiology. **(A)** Partial hepatic ischaemia model with bile duct isolation (Left). Schematic representation of groups in both studies (Right). (**B)** Comparison of bile flow rates (normalised to total body weight). (**C)** Comparison of serum bile acid concentration between the IRI and sham IRI groups. (**D)** TEM images comparing BEC microvilli morphology during early (day 1) and late (day 28) reperfusion timepoints. Data are the mean and standard deviation from 3 separate animals at each time point and treatment, *Significantly different compared to sham IRI group, p<0.05.

A rat model of hepatic IRI was modified from the well described partial (70%) hepatic ischaemia technique [24]. The bile duct was separated from the clamped inflow vessels in order to avoid concomitant cholestatic injury and portal congestion [25] (Fig 1A).

For study 1, animals were divided into IRI and sham IRI groups (n = 21 per group). Anaesthesia was induced using 5% isoflurane delivered via a vaporiser and mixed with oxygen at 5L/min into an anaesthetic chamber. Isoflurane 1–3% was delivered via a facemask to maintain anaesthesia. Upon induction of anaesthesia, animals in both groups received 0.1mg/100gm Meloxicam, 0.05mg/kg Buprinorphine and 150mg/kg Clamoxyl subcutaneously. Lobar vessels supplying the left and middle hepatic lobes were identified and separated from the corresponding bile duct branch. Intra-operative heparin (300IU/kg) was administered intravenously after the vessels were isolated. In the IRI group, these vessels were clamped for 90 min in order to induce lobar IRI. The lobar vessels in the sham IRI group were isolated but not clamped. Apart from clamping, animals in both groups were subjected to similar laparotomy conditions. Subcutaneous Buprinorphine and Clamoxyl were administered 6 hours and then 24 hours postoperatively. Baseline blood samples were taken pre- and postoperatively in both groups. Three animals were randomly selected from each group for each termination timepoint (5 hours, 1, 3, 6, 10, 14 and 28 days post-reperfusion). Anaesthesia was induced and maintained for the terminal laparotomy in a manner similar to the initial procedure. The common bile duct was cannulated to enable bile collection into pre-weighted tubes for sampling and bile flow measurements. Blood samples were also taken and the liver lobes were harvested, weighted and preserved for tissue analysis. To test whether PXR activator treatment modulated liver injury and fibrosis (study 2), rats were divided into IRI-PCN and IRI-vehicle (n = 10 per group) to which PCN (50 mg/kg in 100% DMSO) or vehicle control respectively was administered subcutaneously for 2 days prior to surgery (Fig 1A). Pre-operative analgesia, antibiotic and anaesthetic regimes were administered to animals in both groups on the day of the procedure in a manner similar to that described in the study 1 IRI model. Lobar vessels were identified and isolated from the corresponding bile duct as described in the initial model. The vessels were clamped for 60 minutes in both groups to more closely resemble warm ischaemia in clinical surgery. Intra-operative heparin and postoperative analgesia, antibiotic and fluid regimes were administered in line with the hepatic IRI model. Baseline blood samples were taken pre- and postoperatively in both groups. Daily doses of PCN or vehicle control were injected subcutaneously in the IRI-PCN and IRI-vehicle groups respectively for up to day 10 postoperatively. Animals in each group were assigned one of two time points for termination (1 or 10 days postoperatively) where they were exposed to a second laparotomy. Bile, blood and liver tissue procurement was performed in a manner similar to that described in the initial IRI model. For study 2, a power analysis a priori based on an expected 50% and 20% reduction in inflammatory and fibrotic markers respectively on day 10 according to previous work calculated that a minimum of 4 animals would be required per group assuming a power of 80% and an alpha level of 0.05. A sample size of 5 animals per group for each timepoint in study 2 in keeping with the National Centre for the Replacement Refinement & Reduction’s guidelines to minimise animal use in such experiments.

### Isoprostane analysis

Approximately 200mg of liver was homogenized in 20mL of 2:1 chloroform:methanol containing 0.005% of butylated hydroxytoluene (BHT), to prevent autoxidation. This was allowed to stand for 1 hour at room temperature under a blanket of nitrogen. 4 mL of 0.9% NaCl was added to the samples, which were then shaken vigorously and centrifuged for ten minutes at 2000 rpm and the aqueous layer discarded. The chloroform layer was dried under nitrogen and re-suspended in 1 mL of methanol containing 0.005% BHT. The sample was incubated with 0.5 mL of 15% aqueous potassium hydroxide under nitrogen for 20 minutes at 37°C to hydrolyse phospholipid esterified isoprostanes, and adjusted to pH 3 with 2% phosphoric acid. 5ng of deuterated internal standard was added to the samples, and isoprostanes extracted using Plexa PCX cation exchange solid phase extraction. Isoprostane LC–MS/MS analysis was performed using an AB Sciex 5500 Q-Trap mass spectrometer, equipped with a Turbo V ion spray source operating in negative electrospray ionisation (ESI) mode, coupled to a Shimadzu Prominence UPLC system. Identification and quantification of isoprostanes was performed using multiple reaction monitoring (MRM), monitoring the following transitions: F_2_ isoprostanes, with a parent m/z of 353.2 to product ions with m/z of 193.2, 127.2 and 115.1 for the F_2_-III, IV and VI isomers respectively, and E_2_ isoprostane with a parent m/z of 351.2 to product ions of m/z of 271.2. Chromatographic separation was performed using an Accucore RP-MS column, 50mm x 2.1mm x 2.6μm (Thermo Scientific), and a water/methanol gradient elution from 90% water/0.1% formic acid to 95% methanol/0.1% formic acid over a 20 minute time period at a column flow of 400μL/min. Both the mass spectrometer and LC system were controlled using Analyst 1.5.2 software. Quantitation was performed using standard isotope dilution techniques and Multiquant 2.1software (Sciex).

### Tissue processing and analysis

Formalin fixed samples were embedded in paraffin blocks from which sections (5μm thick) were prepared for staining. For transmission electron microscopy (TEM) imaging, liver samples (less than 2 mm^3^) were fixed in 2% gluteraldehyde in 0.1M sodium cacodylate buffer and stored overnight at 4°C. Samples were subsequently post fixed in 1% osmium tetroxide, dehydrated, embedded in epoxy resin and cut to ultrathin sections (70nm).

Tissue homogenates were prepared by suspending samples in cell lysis buffer (100 mg/ml) and homogenising the tissue manually using a plastic pestle. Samples were then sonicated and centrifuged. Supernatants were extracted and stored at -80°C for later analysis.

Total RNA was extracted from frozen samples homogenised in TRIzol according to the manufacturer’s instructions and quantified using a NanoDrop 2000 spectrophotometer (Thermo Scientific). RNA samples were subsequently treated with DNAse I (Promega) to remove any contaminating genomic DNA.

### Tissue staining

Liver sections were stained with haematoxylin and eosin or Sirius red as previously described [26]. Immunohistochemical staining of paraffin sections was performed using standard HRP-based protocols [27,28]. TEM Sections were stained with 2% uranyl acetate and lead citrate. Sections were then visualised using a Philips CM100 Transmission electron microscope (Philips/FEI Corporation, Eindhoven, The Netherlands) and images stored for further analysis.

### Tissue analysis

Stained slides were scanned using a slide scanner (Leica SCN400) and analysed using SlidePath Digital Image Hub and Tissue IA software (Leica microsystems, Weltzlar, Germany). Morphometric measurements of TEM images were performed using ImageJ analysis software (U.S. National Institutes of Health, Bethesda, Maryland, USA) using a scale bar plugin for microscopes. Biliary epithelial cell (BEC) microvillar density was measured by dividing the total number of luminal microvilli by the circumference of the bile ducts lumen in each cross section. Inflammatory cell counts in H&E sections were normalised to the size of portal or central vein in each field of view as previously described [11]. Liver damage severity was graded on a scale from 0–5 as previously described [27]. For all morphometric and structural histological analysis, slides were assessed independently by two investigators blinded to the treatment groups and measurements were taken in at least nine portal tracts per specimen.

### Electrophoresis, immunoblot analysis and enzyme-linked immune sorbent assay (ELISA)

Protein samples were quantified using the Lowry method [29]. SDS-PAGE was performed using the NuPAGE gel system and immunoblotting was performed using the iBlot dry blotting system as previously described [27,28]. Chemi-luminescent bands were visualised using a G:BOX CCD camera (Syngene, Cambridge, UK) and band densities were quantified using the GeneTools analysis software supplied with the camera. Hyaluronan levels were quantified using an ELISA kit purchased from R&D Systems in accordance with the protocol supplied by the manufacturer.

### Chemokine arrays

Cytokine levels were quantified using multiplex cytokine magnetic bead panel (Millipore, Hertfordshire, UK) as previously described [30]. Fluorescence was detected on a MagPix system and the results analysed using xPONENT software provided with the system.

### Real time qRT-PCR

Quantitative analysis of mRNA expression was performed using SYBR green-based RT-PCR using primer sequences as previously described [31]. RT-PCR was performed using an Applied Biosystems 7500 Fast Real-Time PCR System. 18S rRNA was used as reference gene. The following primer sequences not previously used, were employed to specifically amplify the following nuclear receptor cDNAs: PXR (NM_052980.2) forward primer–aagaacagcaggcgctga and reverse primer–ctgtgaaacaccgcaggtag generating a 106bp product; FXR (NM_021745.1) forward primer–taccattacaacgcgctcac and reverse primer–gcccccgttcttacacttg generating a 93bp product; CAR (NM_022941.4) forward primer–agccacgggctatcatttc and reverse primer–tcttgctgactgttcgtctga generating a 74bp product.

### Thiobarbituric acid reactive substances (TBARS) assay

BHT (5% w/v in methanol) was added to liver homogenate samples during sample processing to minimise ex-vivo lipid peroxidation. The TBARS assay was performed as previously described [32]. The absorbance of supernatants was read at 532nm and TBARS levels in samples were calculated against the calibration curve of MDA standards.

### Serum Chemistry

Serum bile acid levels were quantified using an enzymatic cycling method (Dialab, Wiener Neudorf, Austria) using the manufacturer’s protocol. The total bile acid concentration was derived from the average change in absorbance per minute using a standard formula. Serum alanine aminotransferase (ALT), alkaline phosphatase (ALP) and γ-glutamyltransferase (GGT) were measured as previously described [27].

### Statistical analysis

Results are expressed as mean ± standard deviation (SD) unless stated otherwise. Means were compared using the Student t-test or the ANOVA test (where more than two groups were compared). Tukey’s test was used for post-hoc analysis for significant ANOVA results. Test results with p values of less than 0.05 were accepted as statistically significant. All statistical tests were performed using the Statistical Package for Social Sciences version 19.0 (SPSS Inc., Chicago, IL, USA) and Microsoft Office Excel 2010 (Microsoft Corp., Redmond, WA).

## Results

### IRI caused a transient cholestasis in the absence of bile duct occlusion

To establish whether an acute IRI could result in pathological changes relevant to those observed in DCD liver grafts, rats were subjected to IRI, allowed to recover and the liver examined up to 28 days later (Fig 1A, study 1). Fig 1 demonstrates that rats subjected to 90 minutes of IRI experienced transient cholestasis in the absence of physical clamping of the bile duct, resulting in significant transient reductions in bile flow (Fig 1B) and elevations in serum bile acids (Fig 1C), both of which normalised between 1 and 3 days after the IRI. This injury was associated with a reduction in BEC microvillar density in the ischaemic lobe at the height of cholestasis, although this was reversed due to a compensatory increase in BEC microvillar density by 28 days (Fig 1D), suggestive of a long term alteration in bile duct physiology.

### Liver lobes subjected to IRI experienced high levels of centrilobular and mid-zonal hepatocellular injury, marked ductular reactions, elevations in isoprostane E2 levels and prolonged peri-portal inflammation

**Fig 2:**
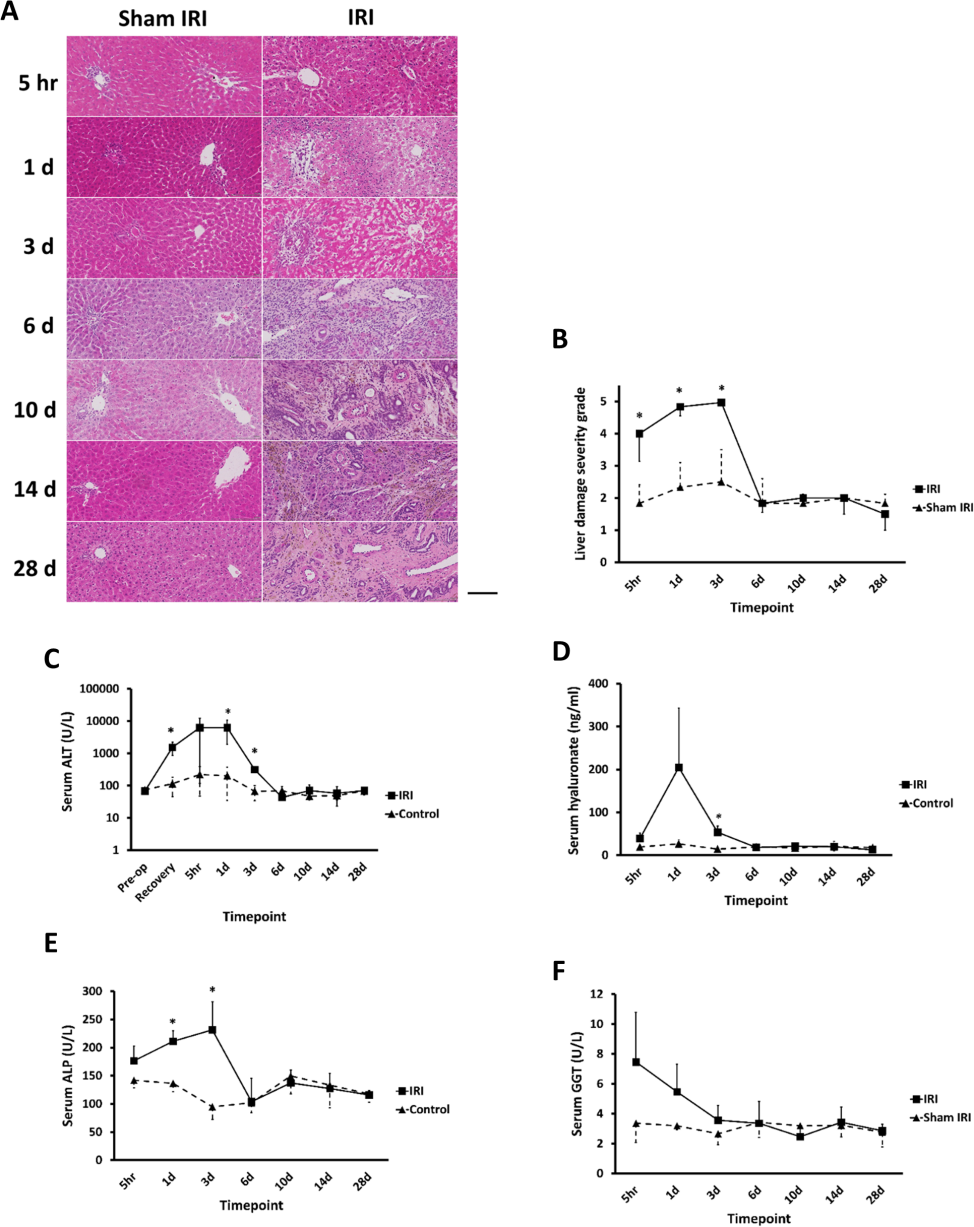
IRI results primarily in centrilobular and mid-zonal hepatocellular injury in addition to sinusoidal and BEC injury. **(A)** H&E staining of liver sections–typical views from the indicated treatment group and time point. Scale bar represents 100μm. (**B)** Comparison of liver damage severity scores between IRI and sham IRI groups assessed on H&E slides (scale of 0–5: normal–extensive damage [9]). (**C-F)** Serum ALT, hyaluronate, ALP and GGT levels respectively for the indicated groups. Data are the mean and standard deviation from 3 separate animals at each time point and treatment, *Significantly different compared to sham IRI group, p<0.05.

Histological examination of liver tissue from ischaemic and non-ischaemic lobes in both the IRI and sham IRI groups demonstrated that IRI resulted in marked ischaemic lobe-specific changes (Fig 2A, see also S1 Fig for lower magnification views). Non-ischaemic lobes from the IRI group and sham IRI groups appeared histologically normal and therefore only sham IRI sections are shown Fig 2A indicates that there was spotty hepatocellular necrosis in zones 2 and 3 evident as early as 5 hours after clamp release, which became more pronounced by day 1. Blinded examination severity scores as defined by Marek et al [9] suggest that liver injury severity was significantly higher in the IRI group up to day 3 post clamp release compared to sham IRI, but that evidence for injury had resolved between day 3 and 6 after clamp release (Fig 2B). These data are supported by serum liver enzymes levels which are suggestive of primarily hepatocellular, but also sinusoidal cell and BEC injury that returned to normal levels between day 3 and 6 after clamp release (Fig 2C–2F).

**Fig 3:**
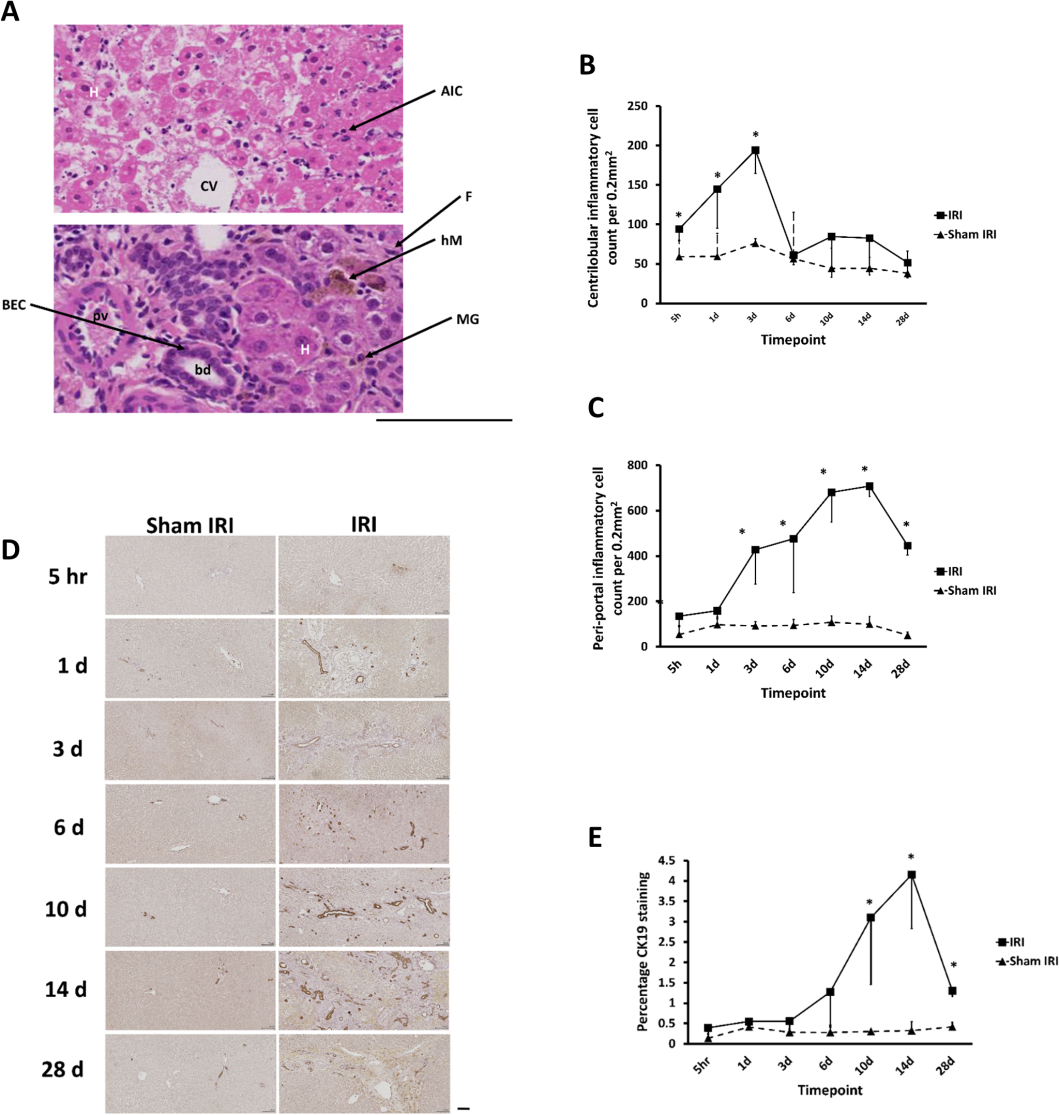
IRI results in persistent inflammatory changes in peri-portal regions and a ductal reaction. **(A)** H&E stained liver sections of a zone 3 (upper) and zone 1 (lower) region in an IRI lobe (day 1 and day 10 respectively):- BEC, biliary epithelial cell; pv, portal venule; bd, bile ductile; AIC, acute inflammatory cell; H, hepatocyte; F, fibroblast or myofibroblast; hM, haemosiderin-laden macrophage; MG, mononuclear granulocyte. Scale bar represents 100μm. **(B-C)** Comparison of inflammatory cell counts in peri-portal and centrilobular areas between the IRI and sham IRI groups. (**D**), cytokeratin 19 (CK-19) staining of liver sections–typical views from the indicated treatment group and time point. Scale bar represents 100μm. E, quantification of CK-19 staining. Data are the mean and standard deviation from 3 separate animals at each time point and treatment, *Significantly different compared to sham IRI group, p<0.05.

A focus on inflammatory cells indicated that there was a progressive inflammation manifest in zone 1 regions (peri-portal) in IRI lobes 5 hours after clamp release that peaked at day 14 (Figs 2A and 3A). Peri-portal inflammatory infiltrates at delayed timepoints contained increasing numbers of mononuclear granulocytes and haemosiderin-laden macrophages. In contrast, inflammation in zone 3 (centrilobular) IRI lobes was largely composed of acute inflammatory cells and was maximal on day 3 after clamp release, returning to baseline levels by day 6 (Figs 2A and 3A–3C). The inflammatory changes in zone 1 were associated with a ductular reaction, neovascularisation and fibrosis from day 3 and led to distortion of the intrahepatic architecture by day 14. The pathological changes in the peri-portal regions persisted for at least 28 days post-reperfusion (Figs 2A and 3A–3C) and were paralleled by ductal reactions in the IRI lobe (Fig 3D and 3E).

**Fig 4:**
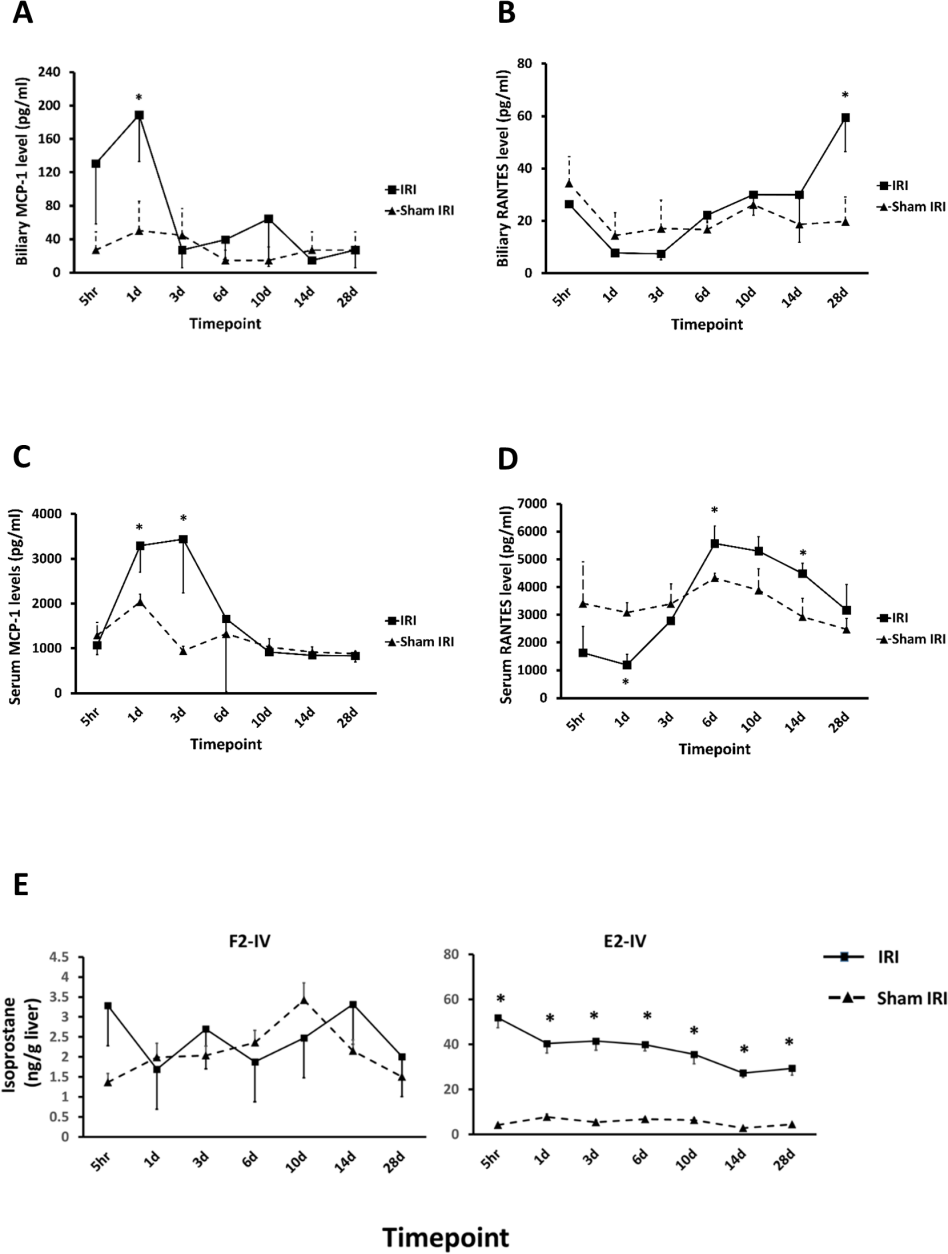
IRI results in increases in biliary and serum cytokine expression. (**A-B)** Comparison of biliary MCP-1 and RANTES concentrations between IRI and sham IRI groups. (**C-D)** Comparison of serum MCP-1 and RANTES levels between IRI and sham IRI groups. (**E)** Comparison of the isoprostane F2 IV (left) and E2 (right) levels in the ischaemic lobe of IRI and sham IRI livers. Data are the mean and standard deviation from 3 separate animals at each time point and treatment, *Significantly different compared to sham IRI group, p<0.05.

To assess the extent of peri-biliary inflammation, cytokine concentrations in bile were examined. Fig 4A and 4C indicates that IRI resulted in an increase in biliary MCP-1 within 5 hours of clamp release, reaching a peak on day 1. Other cytokines, including VEGF, showed a similar rise in the early postoperative period (data not shown). In contrast, biliary levels of RANTES gradually rose from 14 days after clamp release (Fig 4B). The corresponding serum levels of MCP-1 and RANTES are shown for comparison (Fig 4C and 4D).

Isoprostanes are generated from the oxidation of lipids and have been shown to have a potent effect on a variety of biological endpoints [33]. Fig 4E demonstrates that the levels of isoprostane F2-IV between IL and NIL (and similar results were observed for isoprostanes F2-III and VI, data not shown). In contrast, there was a marked elevation in the levels of isoprostane E2 in the IL within 5 hours of IRI compared to the NIL and this elevation persisted for at least 28 days after injury.

### IRI resulted in progressive fibrosis

**Fig 5:**
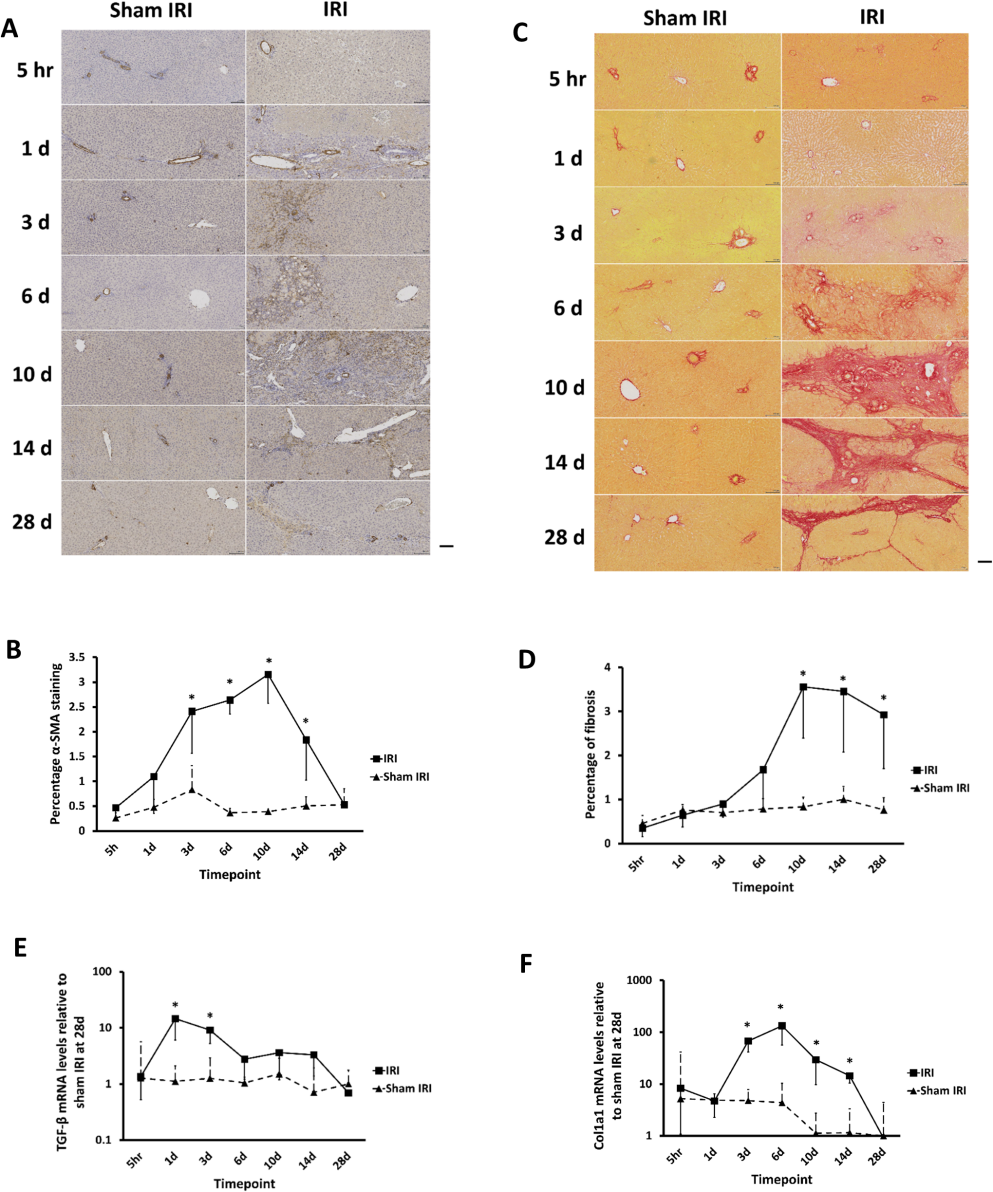
IRI results in progressive fibrosis. **(A)** α-SMA immunohistochemistry—typical views from the indicated treatment group post and time point (upper panels) and quantification of α-SMA immunohistochemistry staining, scale bar represents 100μm. (**B)** quantification of α-SMA staining. **(C)** Sirius red staining–typical views from the indicated treatment group post and time point (upper panels) and quantification of Sirius red staining, scale bar represents 100μm. (**D**) quantification of Sirius red staining. **(E-F)** qRT-PCR analysis in ischaemic and sham ischaemic lobes. Data are the mean and standard deviation from 3 separate animals at each time point and treatment, *Significantly different compared to sham IRI group, p<0.05.

Fig 5A indicates that there was an expansion in the number of α-smooth muscle actin (α–SMA) positive myofibroblasts in the ischaemic lobes in the IRI liver, with a significant increase by day 3 after clamp release. Staining of liver sections for vimentin demonstrated comparable results (S2A and S2B Fig). Since both myofibroblasts and fibroblasts are associated with liver fibrogenesis [34], the extent of fibrosis was assessed by staining liver sections from ischaemic and sham ischaemic lobes with Sirius red. This analysis revealed severe and progressive peri-portal fibrosis in ischaemic lobes and bridging portal tracts at later time points persisting for at least 28 days after the initial injury (Fig 5C and 5D). Non-ischaemic lobes from the IRI group and sham IRI groups appeared histologically normal and therefore only the sham IRI is shown. To confirm these findings, mRNA transcript levels for the pro-fibrogenic cytokine TGF-β and pro-alpha1(I) collagen in ischaemic and sham ischaemic liver lobes were determined by qRT-PCR, using sham ischaemic liver lobes at day 28 as reference samples. TGF-β mRNA transcript levels were significantly higher in the ischaemic lobes between day 1 and 3 post clamp release compared to the sham ischaemic lobes (Fig 5E). Col1A1 mRNA was significantly elevated in the ischaemic lobes in IRI group between day 3 and 14 after clamp release (Fig 5F).

### PXR activation reduced IRI-associated oxidative stress, cholestasis, peri-portal inflammation and fibrosis

To test this hypothesis that drug-mediated PXR activation will have a significant beneficial impact on liver function after IRI through its growth-promoting, anti-inflammatory and anti-fibrotic effects and its promotion of endobiotic excretion, rats were subjected to IRI with or without administration of the rodent-specific PXR activator PCN (Fig 1A, study 2).

**Fig 6:**
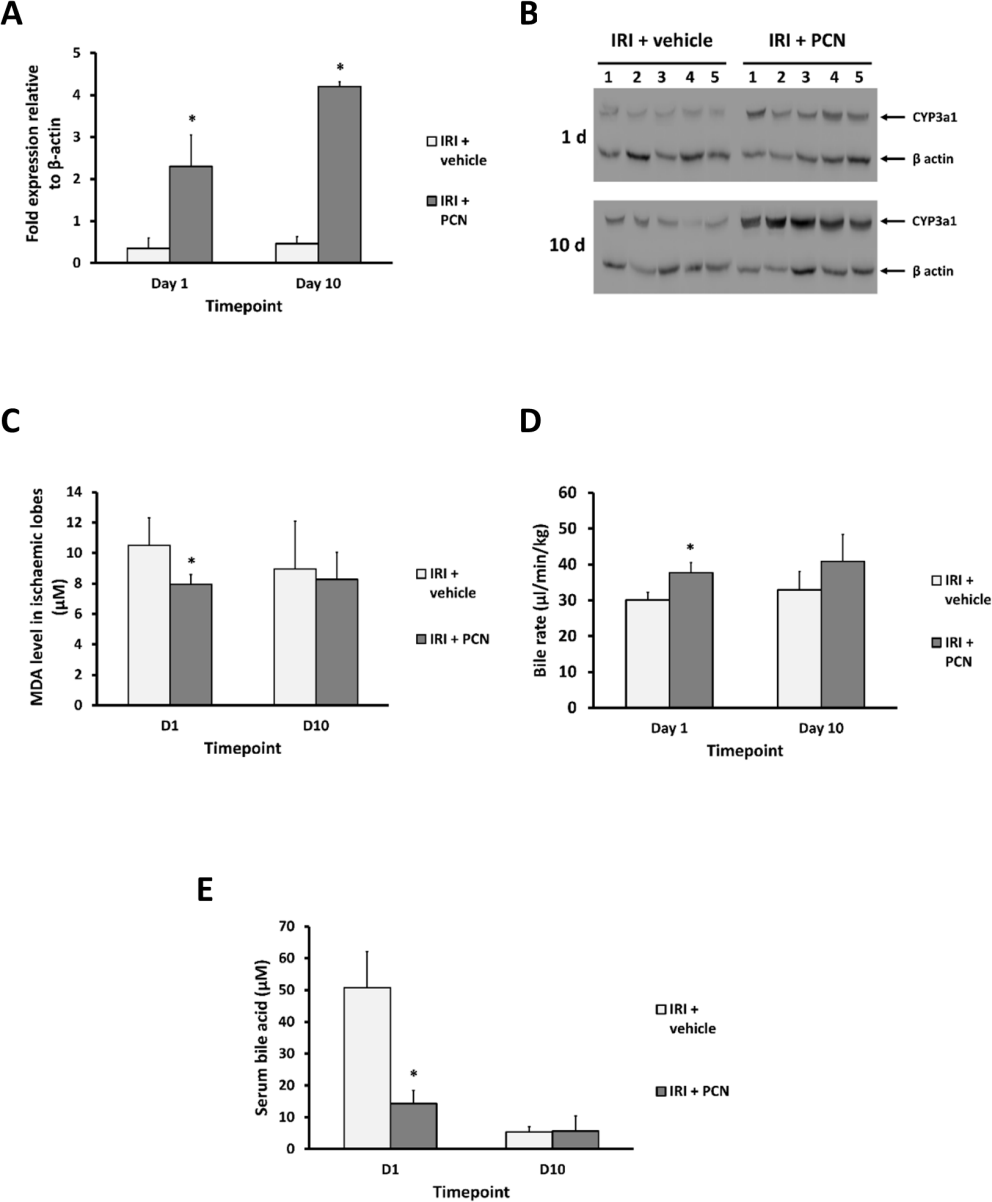
PCN treatment results in hepatic Cyp3a1 induction, reduced oxidative stress and reduced cholestasis in IRI. **(A)** Quantification of Cyp3a1 mRNA levels as determined by qRT-PCR RNA was isolated from whole liver as outlined in the methods section. (**B)** Western blot of Cyp3a1 expression in whole liver homogenates on day 1 and 10 in IRI+PCN and IRI+vehicle groups. (**C)** Liver MDA levels on day 1 post clamp release. (**D)** Comparison of bile flow in the IRI+PCN and IRI+vehicle groups. (**E)** Serum bile acid levels. Data are the mean and standard deviation from 5 separate animals at each time point and treatment, *Significantly different compared to sham IRI group, p<0.05.

Daily administration of PCN in this study resulted in a significantly induced level of expression of the Cyp3a1 mRNA (Fig 6A), a gene product that is transcriptionally regulated by the PXR in the liver [14,35]. This response was also translated into a significant induction of Cyp3a1 protein (Fig 6B), demonstrating that hepatic levels of PCN were sufficient to sustain functional PXR activation as early as 48 hours after commencement of daily PCN treatment. The reported reduction in the levels of expression of nuclear receptors such as the PXR (and observed as a tendency for reduced expression of PXR, FXR and CAR mRNAs, though rarely statistically significant–see S3 Fig) in inflammatory tissues also expressing the PXR [36–38] therefore did not have a significant biological effect on the functionality of the PXR in the liver in these studies. Fig 6C shows that malondialdehyde (MDA) levels were significantly lower in the ischaemic lobes of the IRI-PCN group on day 1 post-reperfusion whereas there were no significant differences in MDA levels in the non-ischaemic lobes.

In addition, PCN treatment significantly increased bile flow rates on day 1 following IRI by 25% compared to the IRI-vehicle group (Fig 6D). Average bile flow remained higher in the IRI-PCN group on day 10 post-reperfusion but this effect did not reach statistical significance. However, serum bile acid levels were significantly lower in the PCN-treated group on day 1 (Fig 6E).

**Fig 7:**
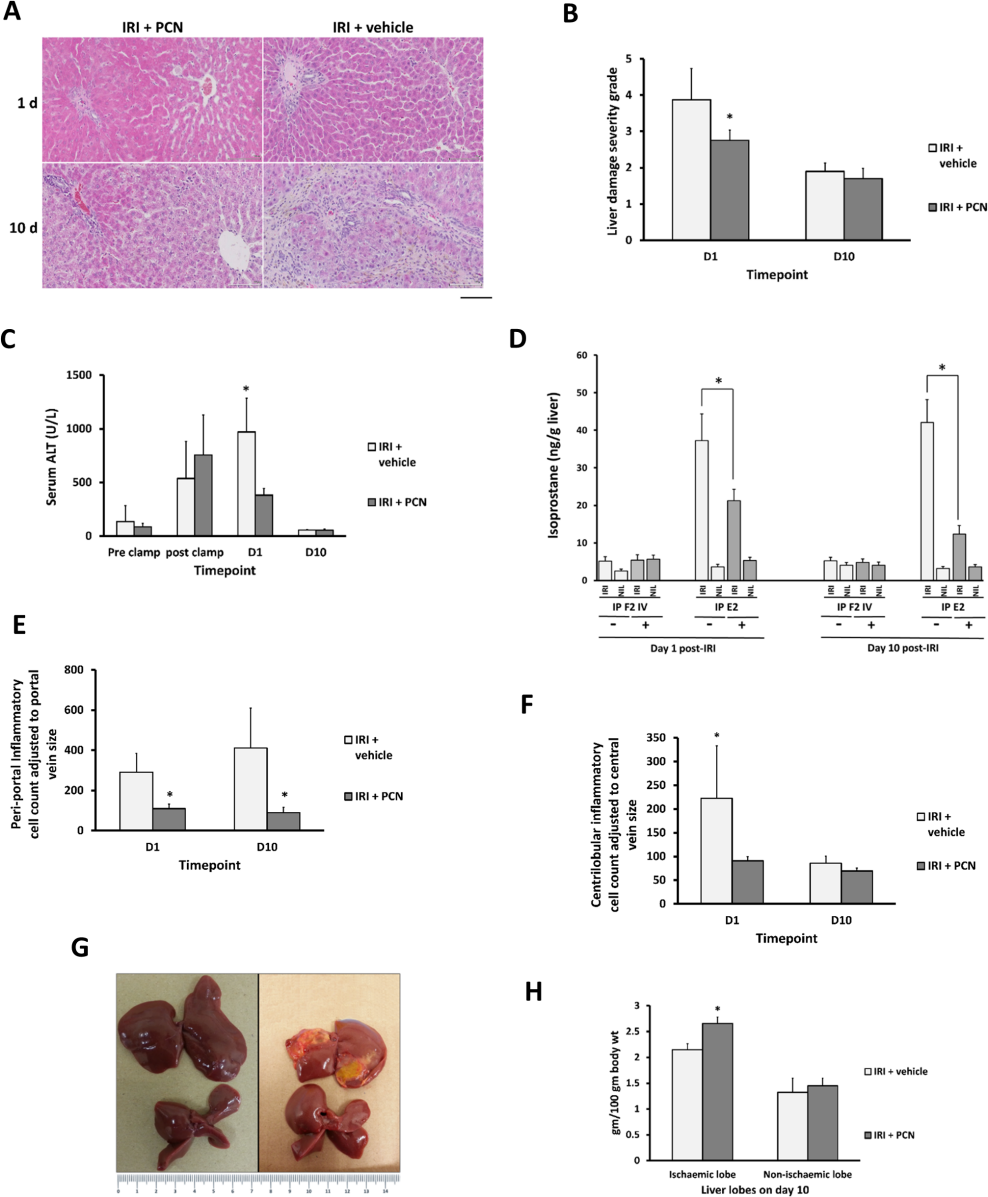
PXR activation reduces hepatocellular injury and peri-portal inflammation associated with IRI, and stimulates liver growth. **(A)** H&E staining of liver sections–typical views from the indicated treatment group post and time point. Scale bar represents 100μm. (**B)** Comparison of liver damage severity scores between IRI + PCN and IRI+vehicle groups assessed [9] on H&E slides (scale of 0–5: normal–extensive damage). (**C)** Serum ALT pre and post-reperfusion. (**D)** Comparison of the isoprostane F2 IV and E2 levels in the ischaemic and non ishaemic lobes from rats subjected to IRI with PCN treatment (+) or DMSO vehicle only treatment (-). (**E-F)** Comparison of inflammatory cell counts in peri-portal and centrilobular areas between the IRI+PCN and IRI-vehicle groups. (**G-I)** Gross changes in size and features of rat liver 10 days following IRI in animals treated with PCN versus vehicle control, non-ischaemic lobes are shown for comparison. Data are the mean and standard deviation from 5 separate animals at each time point and treatment, *Significantly different compared to sham IRI group, p<0.05.

To determine whether these observed PXR-dependent effects on cholestasis and oxidative stress are sufficient to impact on liver injury, the severity of liver damage in both groups was examined. Fig 7A and 7B show that PCN treatment resulted in a significant reduction in liver damage severity scores on day 1 post-reperfusion. Evidence for ongoing necrosis was minimal in both groups by day 10 after IRI in accordance with the findings in study 1. These observations are supported by serum liver enzyme levels which show that ALT levels were significantly lower in the IRI-PCN group on day 1 compared to vehicle control, indicating a significant reduction in hepatocellular injury (Fig 7C). In contrast, serum ALP levels were unaffected by PCN treatment at any time (see S4 Fig). An examination of isoprostane levels in both ischaemic and non-ischaemic lobes demonstrated that the elevated level of isoprostane E2 found in the ischaemic lobe were significantly reduced at both day 1 and day 10 in rats treated with PCN compared to the vehicle control, with no effects seen with isoprostane F2 IV (Fig 7D) or F2 II and VI (data not included). Fig 7E and 7F demonstrate that PCN treatment significantly reduced the number of peri-portal and centrilobular inflammatory cells on day 1 compared to the IRI-vehicle group. The inflammatory cell count in the peri-portal areas remained significantly higher in the vehicle control group on day 10 (Fig 7E). Fig 7G and 7H indicate that PCN treatment also resulted in an increase in the relative wet weight of lobes subjected to IRI at 10 days after clamp release. Recent work has shown that PXR activation alone (by PCN) mediates hepatic hypertrophy but not hyperplasia [21,22]. However, PXR activation by PCN potentiates the hepatic hyperplastic effects of activators of other nuclear receptors (CAR or PPARα) [21,22]. Although it is not possible to determine whether the effect of PCN on the IRI lobe is hypertrophic or hyperplastic, it is most likely that PCN via PXR potentiated a response to the injury in the IRI lobe, since there was no increase in relative wet weight of lobes that were not subjected to IRI.

**Fig 8:**
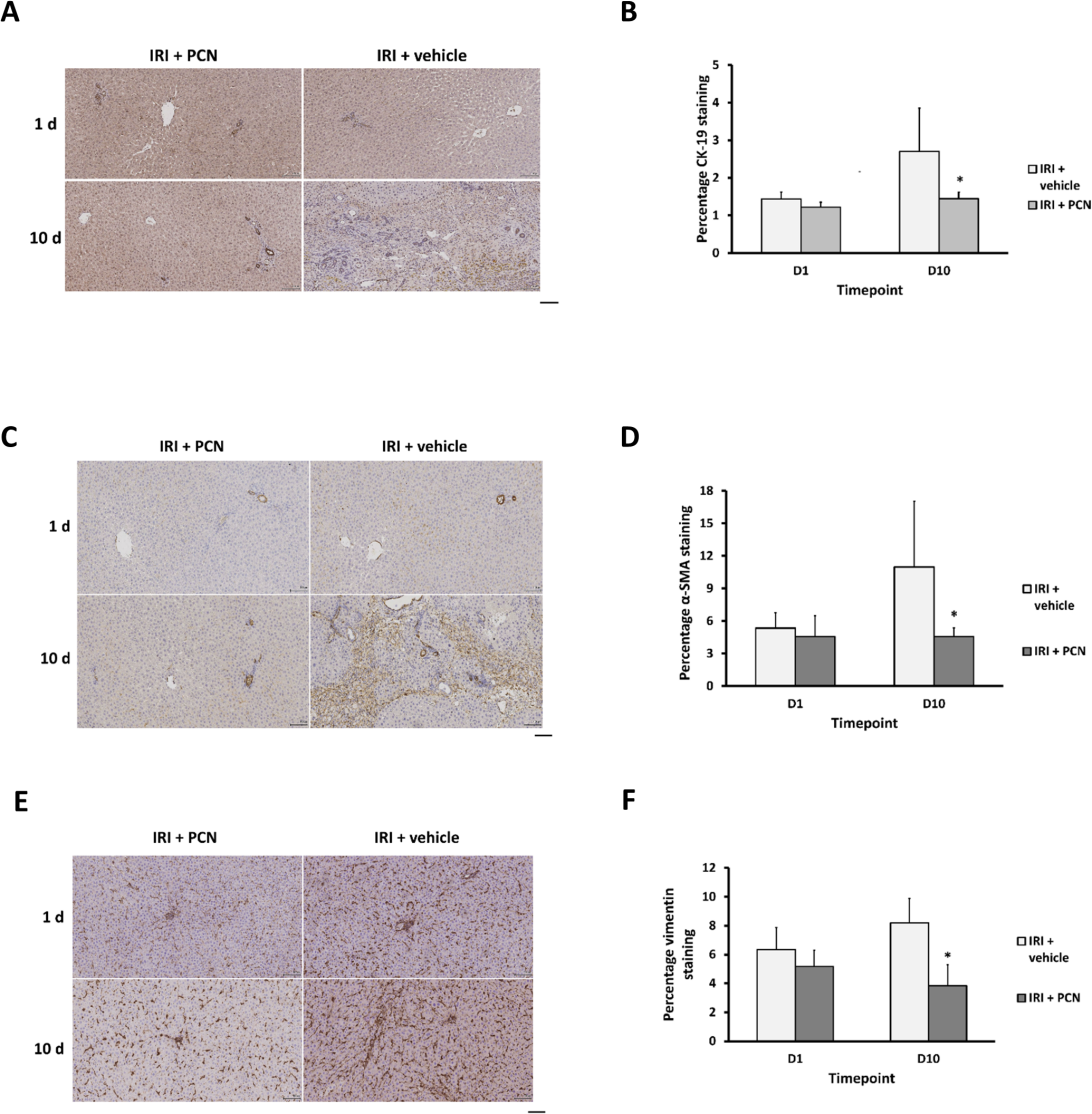
PXR activation reduces IRI-induced ductal reactions and the numbers of fibrogenic cells in the liver. **(A, C, E)** CK-19, α-SMA and vimentin immunohistochemistry respectively between IRI+PCN and IRI+vehicle groups–typical views from the indicated treatment group at the indicated time point after clamp release (scale bar represents 100μm) with (**D, E F)** corresponding stain quantification, data are the mean and standard deviation from 5 separate animals at each time point and treatment, *Significantly different compared to IRI + vehicle group, p<0.05.

An examination of marker endpoints of ductal reactions (CK-19) and fibrosis (α-SMA, vimentin) indicates that PCN treatment reduced the extent of ductal reactions (Fig 8A and 8B) the numbers of α-SMA-positive myofibroblasts (Fig 8C and 8D) and vimentin-positive fibroblasts (Fig 8E and 8F) in peri-portal regions of the liver on day 10 after clamp release.

**Fig 9:**
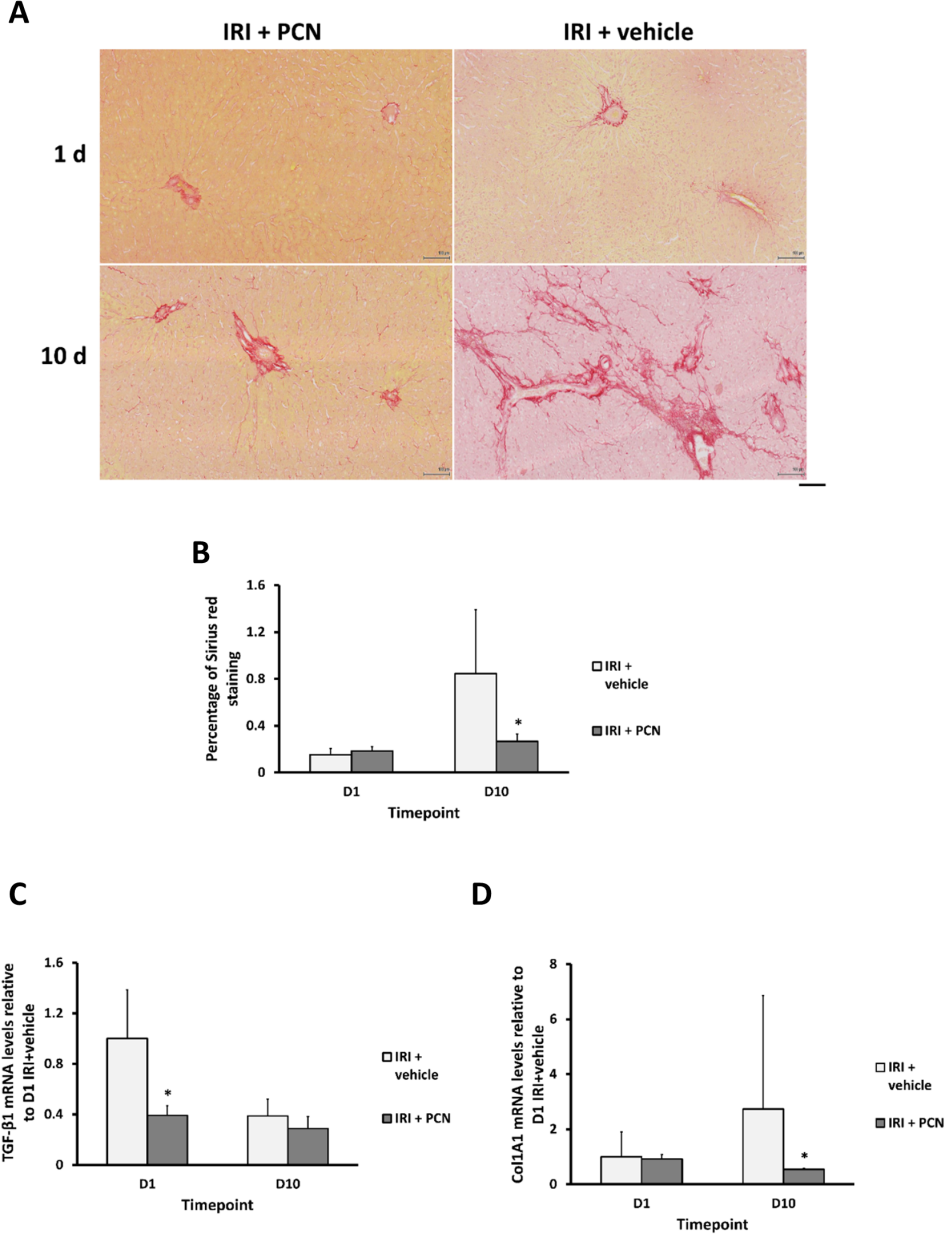
PXR activation reduces IRI-induced fibrosiss in the liver. **(A)** Liver sections stained with Sirius red stain, typical views from the indicated treatment group at the indicated time point after clamp release (scale bar represents 100μm), with (**B)**, corresponding stain quantification. (**C-D)** qRT-PCR analysis for TGF-β1 and Col1A1 mRNA transcripts. Data are the mean and standard deviation from 5 separate animals at each time point and treatment, *Significantly different compared to IRI + vehicle group, p<0.05.

These data are supported by a significant reduction in Sirius red staining of fibrosis in liver sctions (Fig 9A and 9B) and the hepatic levels of mRNA transcripts of TGF-β1 and Col1A1 in the PCN-treated group on day 1 and day 10 respectively following clamp release (Fig 9C and 9D).

## Discussion

The data in this report demonstrate that IRI resulted in marked centrilobular and mid-zonal hepatocellular injury in the early reperfusion period and chronic peri-portal inflammation that persisted for at least 28 days post-reperfusion in the ischaemic lobes, which correlated with specific elevations in isoprostane E2 levels.

Isoprostanes are prostaglandin-like molecules generated by free radical–mediated oxidation of arachidonic acid (and other polyunsaturated fatty acid) moieties in phospholipids, followed by reaction with molecular oxygen and rearrangements to form a variety of structures [33,39]. Isoprostanes are isomeric to prostaglandins, differing in the stereochemical relationships of the two side chains on the prostane ring. After formation, isoprostanes are released from the phospholipid backbone by the action of phospholipases [33,40] or by platelet activating factor/acetylhydrolase [41]. Isoprostanes are therefore generated independently of cyclooxygenases.

The first class of isoprostanes identified were the F2 type isoprostanes [39]. E2 type isoprostane formation is favoured over F2 isoprostanes in tissues when the cellular reducing agents GSH or vitamin E are depleted [42]. They have been shown to be prominent in ischaemic injury in the brain [43]. However, isoprostanes are not terminal products and they have been shown to be readily dehydrated to yield cyclopentone isoprostanes and further metabolised to deoxy-isoprostanes [33, 44]. Free F2 type isoprostanes have been shown to be either metabolised in the liver or to circulate in the plasma and to be renally cleared [45,46]. The data in this paper therefore suggests that the hepatic environment in the ischaemic/re-perfused lobe is oxidising with low cellular reducing agent levels and that the E2 isoprostane generated in response to IRI is either resistant to further hepatic metabolism and/or renal clearance or its generation is sustained by some unidentified mechanism. A marked ductal reaction was also observed, which may have been driven by several factors including transient cholestasis; changes in biliary cell microvilli (although these measurements may have been skewed through normalizing to the circumference of bile ducts as there may be alterations in bile duct size in response to IRI); isoprostane E2 levels; inflammation and/or fibrosis. The associated peri-portal fibrosis also persisted for at least 28 days after clamp release and contrasts with the lack of overt pathological effects observed at this time after acute liver injury by toxins such as CCl_4_ or methapyrilene [26,31,47]. The data in this report also demonstrate that PCN administration enhanced bile flow rates, contributing to a reduction in cholestasis-associated liver injury. This effect of PCN, together with anti-inflammatory, anti-ductal reaction, anti-fibrogenic and growth promoting effects, reduced oxidative stress at early time points as judged by MDA levels (a low-molecular weight aldehyde that can be produced from free radical attack on polyunsaturated fatty acids [48]), early and delayed peri-portal inflammatory cell infiltration, reduced fibrosis and promoted ischaemic lobe hypertrophy and/or hyperplasia following IRI.

The majority of the published research on hepatic IRI in animal models focuses on data within the first few hours/days of reperfusion. However, complications following DCD organ donations that occur subsequent to initial liver injury were the focus of our study. We therefore opted for a 28 day follow up period in study 1 to take into account the delayed clinical presentation of ITBL, a more clinically relevant complication in this group of recipients [5,8]. This has been considered sufficient follow up for long-term complications in an adult rat by previous investigators in view of the relative overall lifespan of adult rats compared to humans [49]. The follow up period in study 2 was based on the time point of maximal fibrosis (10 days) identified in study 1. A modified 70% hepatic IRI model was used in these studies in order to avoid portal congestion associated with proximal hilar inflow occlusion and cholestatic injury associated with concomitant bile duct clamping. Inflow occlusion of 90 minutes in study 1 produced predictable and reproducible damage based on earlier studies in our laboratories using variable occlusion times (data not shown). An ischaemic period of 90 minutes has previously been shown to induce maximal TNF-α production and inflammation [50]. The ischaemic period was reduced to 60 minutes in study 2 in order to more fully represent the clinical situation associated with marginal liver transplants.

This study also shows that activation of the PXR prior to—and after—IRI reduces cellular damage, inflammation and fibrosis. This is consistent with results from previous studies which revealed cyto-protective, anti-inflammatory and anti-fibrotic effects of PXR activation in a number of acute and chronic liver injury models [9,11,13,51–54]. Iannelli et al [12] have previously shown that the administration of clotrimazole—a potent rodent PXR activator—prior to IRI, resulted in an anti-apoptotic effect during the first six hours post-reperfusion. In our study we demonstrate that the cyto-protective effect of PXR activation extends beyond the first few hours post-reperfusion as evidenced by reduced necrosis on haematoxylin and eosin (H&E) stained sections and reduced serum ALT levels on day 1 post-reperfusion when PCN was administered. PXR activation also resulted in a reduction in the degree of lipid peroxidation post-reperfusion. This may be due to the increased expression of PXR-regulated proteins implicated in the oxidative stress response such as glutathione-S-transferase [54]. Reduced oxidative stress may further explain the enhanced cyto-protection associated with PXR activation in addition to the previously described anti-apoptotic effect [12].

In conclusion, this study demonstrates that hepatic IRI results in persistent peri-portal inflammatory and fibrotic changes and that activation of the PXR in the rat prior to and following reperfusion reduces these adverse responses to injury. These findings provide further insight intothe potential role for PXR activation in improving marginal graft outcomes following liver transplantation, if these observations are translatable to man.

## Supporting Information

S1 Fig H&E staining of liver sections in study 1 at lower magnification. Scale bar represents 100μm.
(DOCX)

S2 Fig Comparison of vimentin staining between IRI and sham IRI groups in study 1 (Fig A) with corresponding stain quantification (Fig B). *Significantly different compared to sham IRI group, p<0.05.
(DOCX)

S3 Fig qRT-PCR for nuclear receptor gene expression. qRT-PCR analysis for PXR (**Fig A**), FXR (**Fig B**) and CAR (**Fig C**) transcript levels in study 1. Data are the mean and standard deviation from 3 separate animals at each time point and treatment, *Significantly different compared to sham IRI group, p<0.05. qRT-PCR analysis for nuclear receptor transcript levels in study 2 (**Fig D**). Data are the mean and standard deviation from 5 separate animals at each time point and treatment, ^*^Significantly different compared to IRI + vehicle group, p<0.05.
(DOCX)

S4 Fig ALP levels in study 2. Data are the mean and standard deviation from 5 separate animals at each time point and treatment, ^*^Significantly different compared to sham IRI group, p<0.05.
(DOCX)
