# Supplementary material for: Sustained Isoprostane E2 Elevation, Inflammation and Fibrosis after Acute Ischaemia-Reperfusion Injury Are Reduced by Pregnane X Receptor Activation
Source: PLoS One. 2015 Aug 24;10(8):e0136173. doi: 10.1371/journal.pone.0136173 (PMC4547732; doi:10.1371/journal.pone.0136173)

**Supporting information Supp. Figure 1: H&E staining of liver sections in study 1 at lower magnification**. Scale bar represents 100μm.


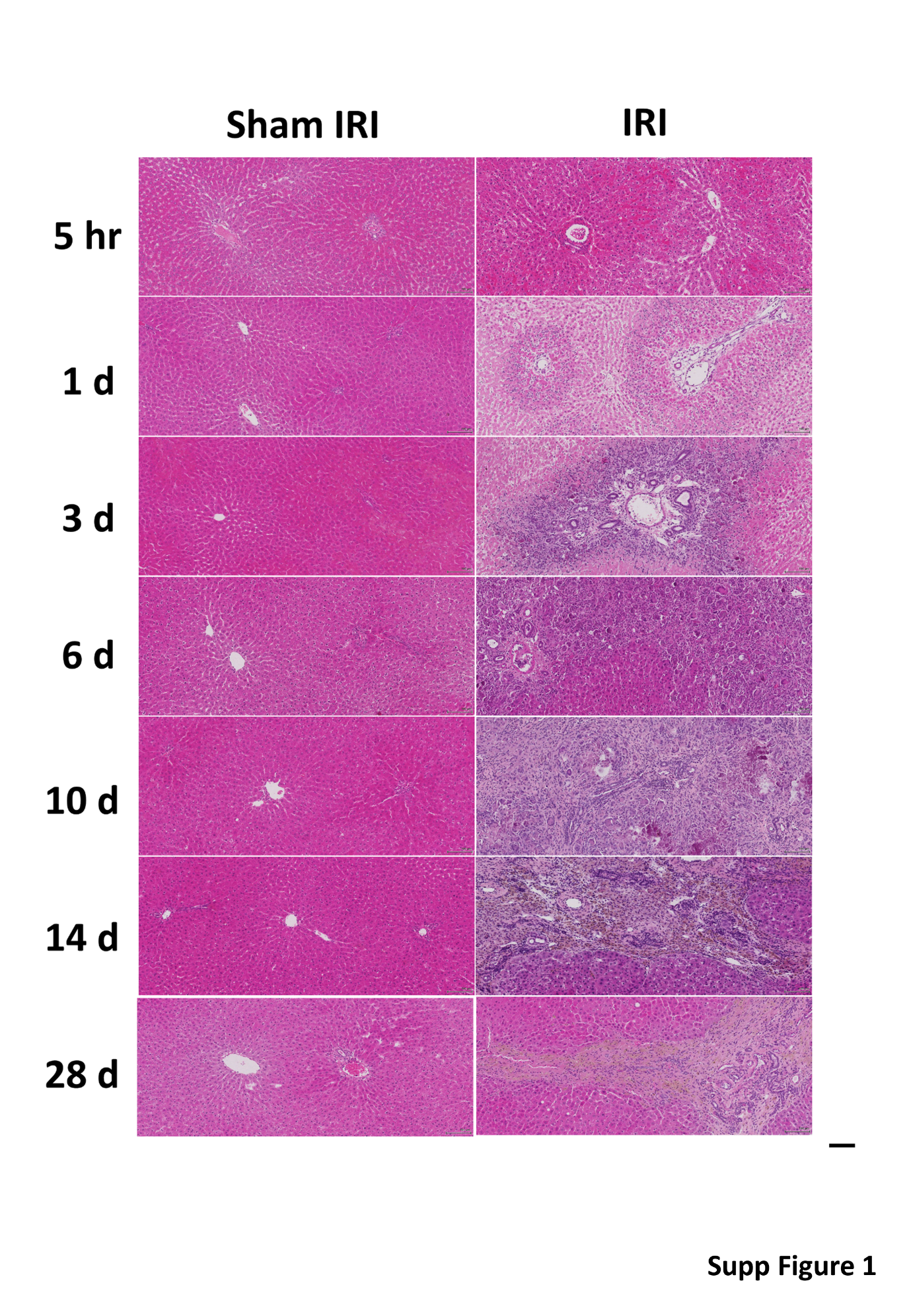

Supplement: S1 Fig — Scale bar represents 100μm. (DOCX) [file pone.0136173.s001.docx]
