# Supplementary material for: Sustained Isoprostane E2 Elevation, Inflammation and Fibrosis after Acute Ischaemia-Reperfusion Injury Are Reduced by Pregnane X Receptor Activation
Source: PLoS One. 2015 Aug 24;10(8):e0136173. doi: 10.1371/journal.pone.0136173 (PMC4547732; doi:10.1371/journal.pone.0136173)

**Supporting information Supp. Figure 2: Comparison of vimentin staining between IRI and sham IRI groups in study 1 (A) with corresponding stain quantification (B).** ^*^Significantly different compared to sham IRI group, p<0.05.


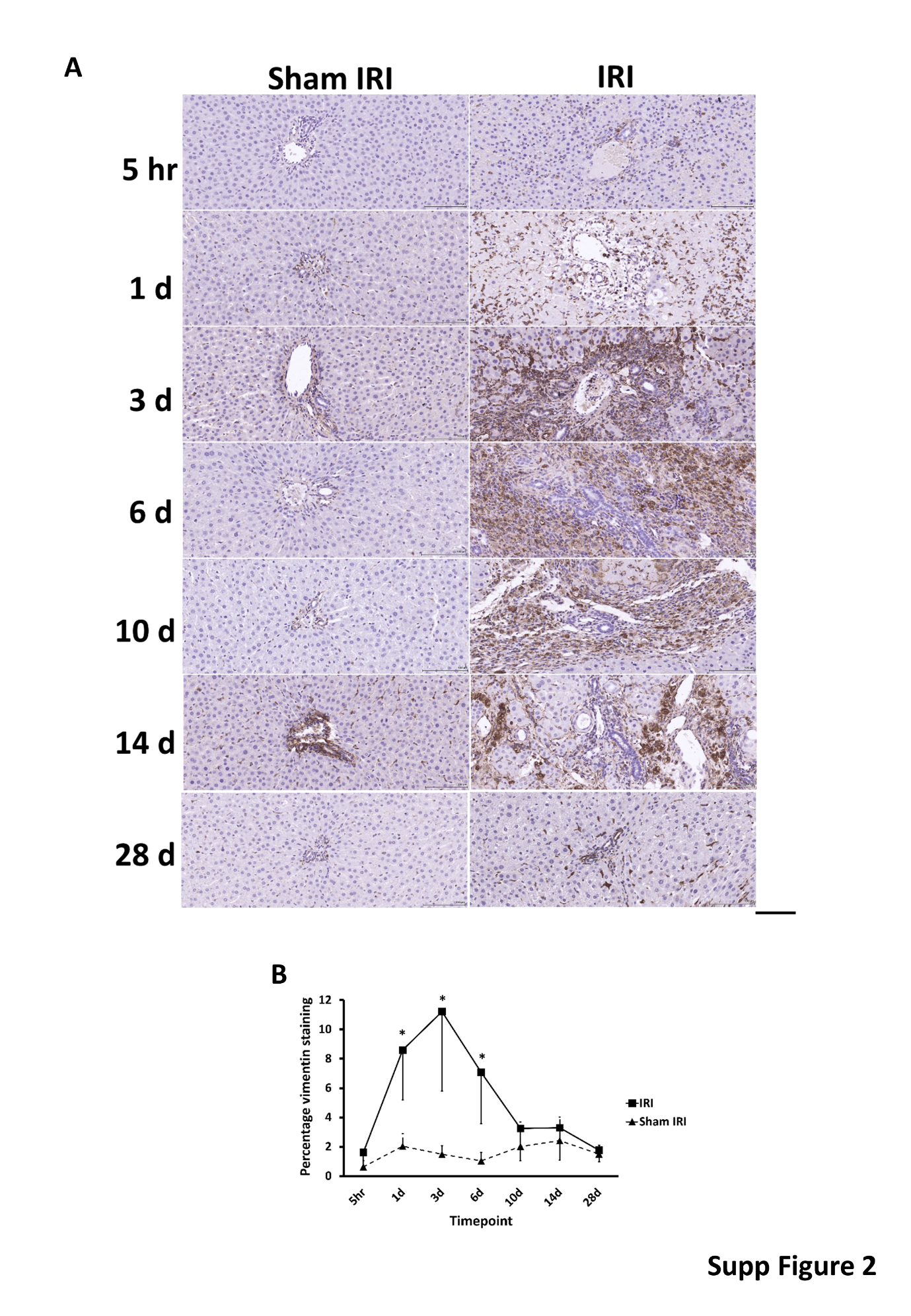

Supplement: S2 Fig — *Significantly different compared to sham IRI group, p<0.05. (DOCX) [file pone.0136173.s002.docx]
