# Supplementary material for: Sustained Isoprostane E2 Elevation, Inflammation and Fibrosis after Acute Ischaemia-Reperfusion Injury Are Reduced by Pregnane X Receptor Activation
Source: PLoS One. 2015 Aug 24;10(8):e0136173. doi: 10.1371/journal.pone.0136173 (PMC4547732; doi:10.1371/journal.pone.0136173)

**Supporting information Supp. Figure 3: qRT-PCR for nuclear receptor gene expression.** qRT-PCR analysis for PXR (**A**), FXR (**B**) and CAR (**C**) transcript levels in study 1. Data are the mean and standard deviation from 3 separate animals at each time point and treatment, *Significantly different compared to sham IRI group, p<0.05. **D**: qRT-PCR analysis for nuclear receptor transcript levels in study 2. Data are the mean and standard deviation from 5 separate animals at each time point and treatment, ^*^Significantly different compared to IRI + vehicle group, p<0.05.


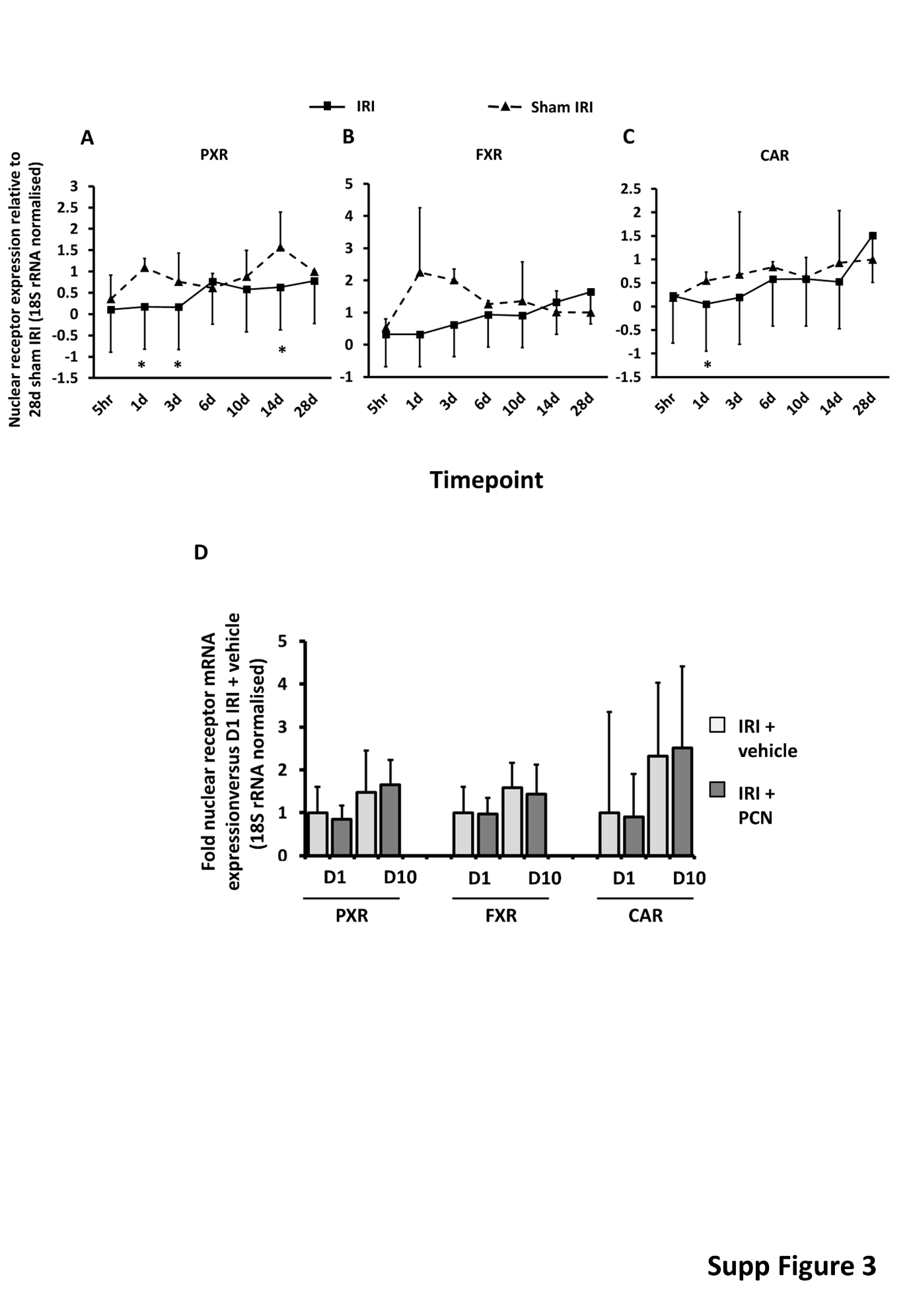

Supplement: S3 Fig — qRT-PCR analysis for PXR (Fig A), FXR (Fig B) and CAR (Fig C) transcript levels in study 1. Data are the mean and standard deviation from 3 separate animals at each time point and treatment, *Significantly different compared to sham IRI group, p<0.05. qRT-PCR analysis for nuclear receptor transcript levels in study 2 (Fig D). Data are the mean and standard deviation from 5 separate animals at each time point and treatment, *Significantly different compared to IRI + vehicle group, p<0.05. (DOCX) [file pone.0136173.s003.docx]
