# Supplementary material for: Sustained Isoprostane E2 Elevation, Inflammation and Fibrosis after Acute Ischaemia-Reperfusion Injury Are Reduced by Pregnane X Receptor Activation
Source: PLoS One. 2015 Aug 24;10(8):e0136173. doi: 10.1371/journal.pone.0136173 (PMC4547732; doi:10.1371/journal.pone.0136173)

**Supporting information Supp. Figure 4: ALP levels in study 2.**  Data are the mean and standard deviation from 5 separate animals at each time point and treatment, ^*^Significantly different compared to sham IRI group, p<0.05.


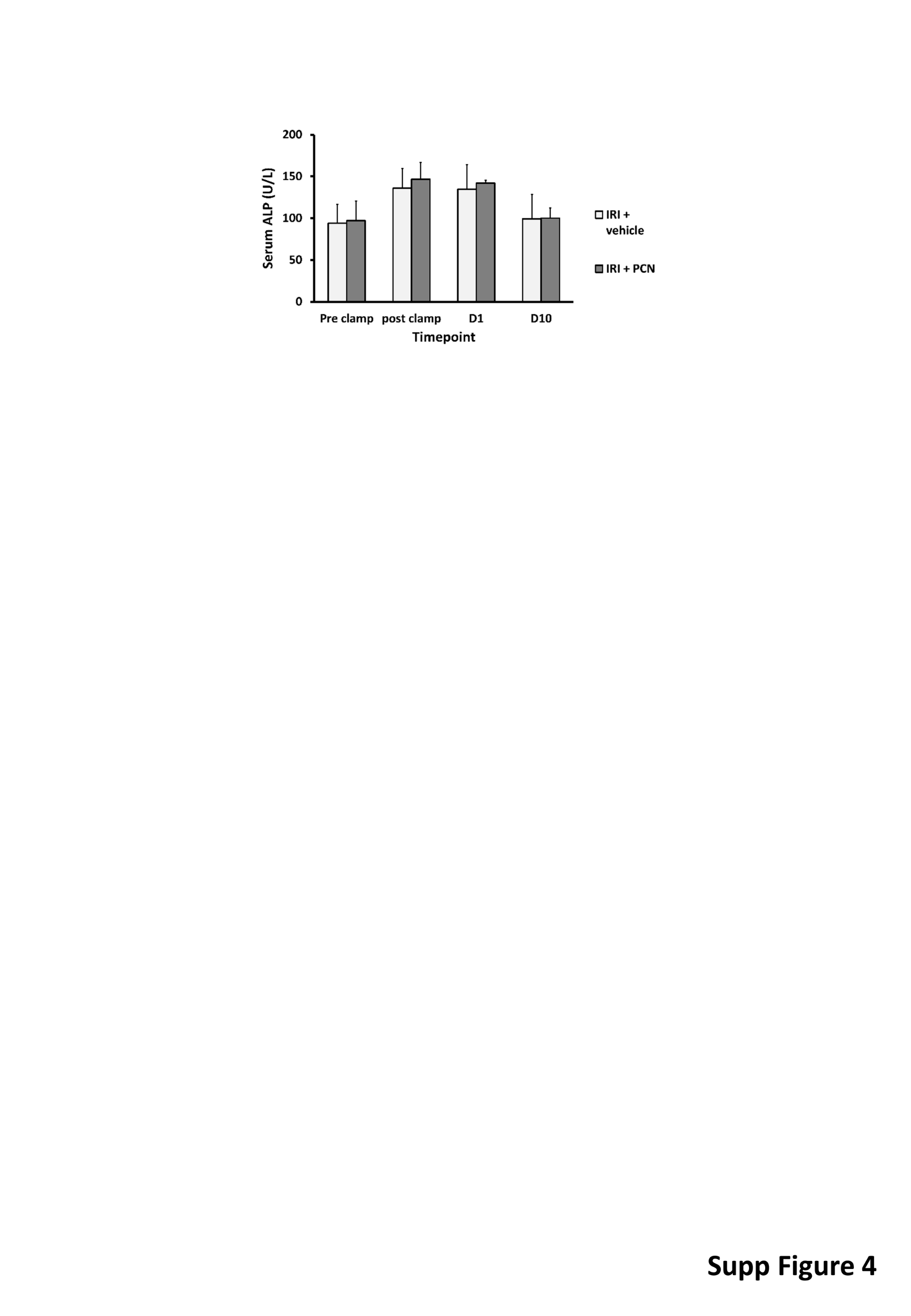

Supplement: S4 Fig — Data are the mean and standard deviation from 5 separate animals at each time point and treatment, *Significantly different compared to sham IRI group, p<0.05. (DOCX) [file pone.0136173.s004.docx]
